# Phase Transition of Huntingtin: Factors and Pathological Relevance

**DOI:** 10.3389/fgene.2020.00754

**Published:** 2020-07-23

**Authors:** Junsheng Yang, Xiaotong Yang

**Affiliations:** Collaborative Innovation Center of Yangtze River Delta Region Green Pharmaceuticals, College of Pharmaceutical Sciences, Zhejiang University of Technology, Hangzhou, China

**Keywords:** huntingtin, phase separation, phase transition, aggregates, Huntington’s disease

## Abstract

Formation of intracellular mutant Huntingtin (mHtt) aggregates is a hallmark of Huntington’s disease (HD). The mechanisms underlying mHtt aggregation, however, are still not fully understood. A few recent studies indicated mHtt undergoes phase transition, bringing new clues to understand how mHtt aggregates assemble. Here in this mini review, we will summarize these findings with a focus on the factors that affect mHtt phase transition. We will also discuss the possible pathological roles of mHtt phase separation in HD.

## Introduction

Huntington’s disease (HD) is a genetic neurodegenerative disease caused by expanded CAG triplet repeats in the first exon of *HTT* gene (MacDonald et al., 1993). A biological hallmark of HD is the formation of intracellular insoluble protein aggregates (inclusions) found in the brains of affected patients (DiFiglia et al., 1997; Becher et al., 1998; Gutekunst et al., 1999). *In vivo* and *in vitro* studies in the past two decades confirmed that these aggregates are composed of mutant Huntingtin (mHtt) alone or together with other proteins [reviewed in Arrasate and Finkbeiner (2012) and Jimenez-Sanchez et al. (2017)]. This is partially because the expanded CAG repeats in mutated *HTT* translate to an expanded polyglutamine (polyQ) sequence in mHtt and makes it prone to misfolding and aggregating (Scherzinger et al., 1997, 1999). Whether these aggregates found in HD patients are toxic, benign or even protective, however, has been long debated. On the one hand, the correlation between polyQ length, aggregation appearance and HD age-of-onset indicates potential toxicity of mHtt aggregates (Davies et al., 1997; Ordway et al., 1997; Becher et al., 1998; Perutz and Windle, 2001). On the other hand, the formation of mHtt aggregates has also been reported to be separable from cell death and might even be beneficial for cell survival (Saudou et al., 1998; Kim et al., 1999; Arrasate et al., 2004).

While the debate goes on, the field’s understanding of mHtt aggregation and toxicity, nonetheless, has been evolving as well. It is now known that mHtt proteins exist in the forms of monomers, soluble oligomers, and insoluble polymers both *in vivo* and *in vitro*. The formation of mHtt oligomers and polymers from monomers is polyQ length- and time-dependent, likely in a monomer→oligomer→polymer order (Poirier et al., 2002; Iuchi et al., 2003; Takahashi et al., 2008; Lajoie and Snapp, 2010; Legleiter et al., 2010; Olshina et al., 2010; Marcellin et al., 2012; Kim et al., 2016). mHtt species of similar sizes can manifest conformational polymorphism. For monomers and oligomers, mHtt species recognized by the monoclonal antibody 3B5H10 shows higher neurotoxicity than the others (Miller et al., 2011; Peters-Libeu et al., 2012). Structural biology indicates these 3B5H10-recognized mHtt species adopt a more compact conformation. A later work found that they are resistant to selective autophagy and thus have a lower degradation rate (Fu et al., 2017). Whether this is directly linked to their compact structure is not yet clear. Interestingly, the insoluble mHtt polymers/aggregates also have diverse conformations. A study using synchrotron-based infrared microspectroscopy revealed that mHtt aggregates from adult and juvenile HD patients’ brains display three conformations (non-amyloid, amyloid with β-sheet/unordered, and amyloid exclusively with β-sheet), among which the amyloid with β-sheet/unordered structures is distributed positively correlated with brain regions affected by HD (Andre et al., 2013). Another study in immortalized mouse striatal STHdhQ7/Q7 cells found that overexpressed mutant huntingtin fragments can form two types of inclusions, the tightly packed fibrillar inclusion and the loosely packed globular inclusion, based on their morphological appearance under super-resolution microscopy and fluorescence lifetime imaging microscopy (FLIM)-Förster resonance energy transfer (FRET) (Caron et al., 2014). The authors also found that these two types of inclusions have different dynamic properties, in that the fibrillar inclusion does not exchange protein with the soluble phase while the globular inclusion does. Similarly, a later study using an Htt exon1 biosensor that can distinguish protein structures based on their accessibility to biarsenical dyes revealed that mHtt exon1 product (mHtt_ex__1_) forms disordered inclusions early and then convert to β-sheet-rich amyloid over time (Ramdzan et al., 2017). Taken together, accumulating evidence indicates that mHtt forms conformationally distinct monomers, polymers and inclusions (MacDonald et al., 1993); mHtt species with certain conformation(s) are more toxic than the others (DiFiglia et al., 1997); different mHtt conformations may convert from one to another (Becher et al., 1998). The mechanism(s) underlying their formation and possible conversion, however, is still largely unknown.

Very recently, several studies carried out *in vitro* in yeast and mammalian cells indicated that the formation of mHtt assemblies is mediated by phase separation and phase transition (Peskett et al., 2018; Posey et al., 2018; Aktar et al., 2019). These findings suggested the involvement of a new mechanism in the formation of mHtt aggregates and thus provided new clues to understand their polymorphism in conformation and toxicity. We will summarize these works and discuss their possible pathological relevance in the following sections.

## Phase Transition in Cells

**FIGURE 1:**
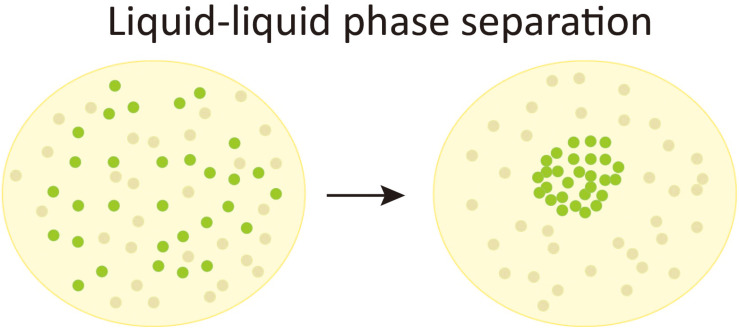
Liquid-liquid phase separation. A schematic chart of liquid-liquid phase separation (LLPS). In a solution composed of evenly distributed solute molecules (the green bubbles) and solvent molecules (gray bubbles), when LLPS occurs, the solute molecules condense to form a membraneless liquid-like phase and leaves a surrounding region absent of the solute molecules.

The term “phase” in physics is used to describe a thermodynamic system composed of materials with uniform physical properties. The change from one phase to another is coined phase transition (e.g., a transition from liquid phase to solid phase). Liquid–liquid phase separation (LLPS) is a special kind of phase transition that refers to the separation of a solution into two distinct co-existing phases, whereas one is solute-enriched and the other is solute-absent (Alberti, 2017) (also see Figure 1). Although the idea that cytoplasm to be a mixture of liquid with suspending droplets of different chemical properties can be tracked back to as early as 1899 (Wilson, 1899), the intracellular phase separation was not experimentally demonstrated until Brangwynne et al. (2009) found that the P granule is liquid-like and formed by LLPS. The same group also revealed that nucleoli also have liquid-like behavior (Brangwynne et al., 2011). During the past decade, the intracellular phase separation of more biological macromolecules has been discovered, suggesting that phase separation might be a common mechanism for cells to regulate essential biological processes [reviewed in Alberti (2017), Gerlich (2017), Shin and Brangwynne (2017), Boeynaems et al. (2018)]. In line with this notion, aberrant phase separation is proposed to contribute to the pathology of diseases (Hyman et al., 2014; Cable et al., 2019; Verdile et al., 2019).

## Phase Transition of mHtt

### mHtt Forms Different Phases and Undergoes Phase Transition

A few recent studies indicate that mHtt undergoes phase transition to form higher-ordered assemblies, both *in vitro* and in cells.

Using a combination of solubility analysis, right-angle static light scattering and transmission electron microscopy, Posey et al. (2018) reported that purified huntingtin N-terminal fragments (Htt-NTF) formed three phases distinguished by their saturation concentration, size, and shape. More specifically, the authors designated them as the M phase (consists of soluble monomers and oligomers), the S phase (mainly consists of bigger soluble aggregate spheres sized about 25 nm in diameter), and the F phase (mainly consists of insoluble fibrillar aggregates). Htt-NTF can separate into different phases when its concentration goes above a construct-specific saturation concentration.

In another study published in 2018, Peskett et al. (2018) used correlative light and electron microscopy (CLEM) and time-lapse fluorescence microscopy to study the aggregation of Htt_ex__1_-GFP proteins. They observed that Htt_ex__1_-GFP forms dim liquid-like and bright solid-like assemblies, *in vitro*, in yeast cells as well as in mammalian cells. More interestingly, the authors also found that the liquid-like Htt_ex__1_-GFP assemblies are formed by LLPS and can convert into solid-like assemblies by phase transition.

Aktar et al. (2019) reported that in yeast cells, mHttex1-GFP form bright membrane-less spherical phase-separated inclusion bodies (IBs) with uniform GFP intensity. However, based on the timescale in FRAP experiments, the authors concluded that the nature of IBs they observed are not “fully liquid” nor solid but rather gel-like. Besides the IBs, the authors also observed clusters of smaller inclusions (which they named cluster like inclusions or CLIs) that are asymmetrical in shape and GFP intensity, in a small proportion of cells. Moreover, the authors also tracked the random movements of small mHtt_ex__1_-GFP particles and propose that their collision and coalescence lead to the development of mHtt inclusions.

Taken together, the above-mentioned studies all indicated that Htt_ex__1_ manifest phase transition, supported by evidence from thermo-kinetics, light scattering, quantitative fluorescent microscopy, and electron microscopy experiments. However, besides the common findings pointing to mHtt phase transition, these studies used quite different methodology and focused on different aspects of mHtt phase behavior (see Table 1 for summary). For instance, Posey et al. (2018) studied sub-micron-sized Htt species and factors that affect their thermo-kinetic properties with biochemical and biophysical approaches, while Peskett et al. (2018) and Aktar et al. (2019) observed micron-sized Htt inclusions and their liquid- or solid-like properties mainly with fluorescent microscopy assisted by quantitative analysis. Considering the diverse sizes and conformations of mHtt species, thorough works with multiple mutually supportive approaches are needed to further explore the phase properties of mHtt assemblies. Nonetheless, these latest studies have shed new light to the understanding of the polymorphism of mHtt species at different scales.

**TABLE 1:** Summary of mHtt phase behavior.

**Htt fragments**	**System**	**Phase behavior**	**References**
mHtt-NTF-40Q	*In vitro*	Concentration dependently forms separable M phase (soluble monomer), S phase (soluble aggregates) and F phase (insoluble fibrillar aggregates)	Posey et al., 2018
mHtt_ex1_-25Q-EGFP	Budding yeast	None	Peskett et al., 2018
mHtt_ex1_-25QP-EGFP	*In vitro* Budding yeast Mammalian cell	LLPS, liquid to solid transition LLPS LLPS	
mHtt_ex1_-43Q-EGFP	Budding yeast	LLPS, liquid to solid transition	
mHtt_ex1_-43QP-EGFP	Budding yeast Mammalian cell	LLPS, liquid to solid transition LLPS, liquid to solid transition	
mHtt_ex1_-97Q-EGFP	Budding yeast	LLPS, liquid to solid transition	
mHtt_ex1_-97QP-EGFP	Budding yeast Mammalian cell	LLPS, liquid to solid transition LLPS, liquid to solid transition	
mHtt_ex1_-25QP-EGFP	Budding yeast	None	Aktar et al., 2019
mHtt_ex1_-72QP-EGFP	Budding yeast	Phase separated to gel-like inclusions	

### Factors Affecting mHtt Phase Transition

Diverse factors are known to affect mHtt aggregation; whether they can also modulate mHtt phase transition was also explored by in the studies discussed in the previous section.

Posey et al. (2018) characterized the saturation concentrations of soluble mHtt-NTF for its S and F phases and found that the sequence flanking the mHtt polyQ region affects the phase boundaries. Specifically, the 17N-terminal amino acids of mHtt (N17) lower the fibrillar-phase saturation concentration *c*_F_, indicating N17 facilitates mHtt-NTF phase separation *in vitro*, while the proline-rich region downstream of polyQ sequence (i.e., P-rich region) increases *c*_F_, suggesting it lowers the driving force for mHtt-NTF phase separation. The authors also studied in depth how profilin, a small actin-binding protein, may contribute to the modulation of mHtt-NTF phase separation. They observed that profilin preferably binds to the soluble monomers and oligomers of mHtt-NTF, thereby destabilize mHtt-NTF aggregates. Moreover, computer simulation suggests profilin interacts with mHtt-NTF through a combined effect of specific binding with the P-rich region and auxiliary binding with the polyQ sequence. Interestingly, mHtt-NTF with longer polyQ (40Q vs. 30Q) showed higher affinity with profilin in protein binding assays.

Peskett et al. (2018) also looked at polyQ length and the P-rich region for their roles in mHtt phase transition. In both yeast and mammalian cells, mHtt_ex__1_ with different length of polyQ (25, 43, and 97) can all form liquid-like assemblies, but a polyQ expansion (43 and 97) is required for solid-like assemblies’ formation. *In vitro*, however, even mHtt_ex__1_ with 25Q can also undergo liquid to solid transition. P-rich region assists the mHtt_ex__1_ to form assemblies in yeast, whether this is also the case in mammalian cells is unclear. The authors also tested whether electrostatic interactions play a role in mHtt phase separation and found that even very high salt concentration (up to 1M) had little effect on mHtt-25QP droplet formation *in vitro*. In their yeast experiments, the authors also looked at the yeast prion [RNQ+] and found it not required for mHtt_ex__1_ phase separation.

Aktar et al. (2019) checked phase transition of mHtt_ex__1_ with different polyQ length (25Q and 72Q) as well. Consistent with previous reports (Krobitsch and Lindquist, 2000; Meriin et al., 2002) and Peskett et al.’s work, mHtt_ex__1_-25Q did not form inclusions. Besides, Hsp104, a yeast disaggregase, is required for inclusion formation as reported before (Krobitsch and Lindquist, 2000; Meriin et al., 2002). Interestingly, Aktar et al. (2019) did not see mHtt_ex__1_-72Q forming any liquid-like or solid-like assemblies in *rnq1*Δ strains.

In summary, several factors can affect mHtt phase transition. Expanded polyQ is required for mHtt phase transition in both studies with mHtt_ex__1_-GFP under fluorescent microscopy. The effect of the flanking sequences, however, looks somewhat controversial. The P-rich region lowers the driving force for phase separation *in vitro* and facilitates profilin in suppressing mHtt_ex__1_ aggregation (Posey et al., 2018), but promotes intracellular mHtt_ex__1_ phase separation in yeast (Peskett et al., 2018). One possible explanation for this discrepancy is, compared with the *in vitro* aggregation system, the intracellular milieu contains many more mHtt-interacting proteins potentially affecting mHtt phase separation. Profilin, Hsp104, and Rnq1 are some examples, although the exact mechanisms by which they modulate mHtt phase separation still requires further studies. It’s worth mentioning that although Peskett et al. (2018) found the [RNQ+] prion is not required for mHtt_ex__1_ phase separation, Aktar et al. (2019) reported yeast cells lacking the RNQ1 gene were unable to form mHtt assemblies. Because these two experiments were carried out in different yeast strains with different [RNQ+] prion-forming properties, it is thus still unclear whether the non-prion form of Rnq1 protein assists mHtt phase separation.

Besides the intrinsic and intracellular factors explored in the above-mentioned studies, there are also factors that may affect mHtt phase transition in intercellular manners. mHtt aggregates were first reported to have prion-like properties such as templated misfolding *in vitro* and intercellular propagation in co-cultured cells [reviewed in Pearce and Kopito (2018)]. Later studies reported that mHtt aggregates also underwent intercellular transfer in mouse brains and contribute to HD pathology phenotypes (Pecho-Vrieseling et al., 2014; Jeon et al., 2016). A more recent work using a FRET-based mHtt aggregate seeding assay indicates a strong correlation between mHtt species’ seeding activity and HD pathology progression (Ast et al., 2018). It is thus crucial to explore whether and how an mHtt aggregate “seed” obtained via intercellular transfer may affect the local mHtt phase transition. In addition, recent studies in Alzheimer’s disease (AD) cells suggest that microglia are capable of uptake tau “seeds” but may promote their intercellular propagation via incomplete degradation (Spanic et al., 2019). Whether similar mechanisms also contribute to mHtt aggregation intercellularly remain to be explored.

### Htt Phase Transition and HD: Pathological Relevance

Although the discovery of phase transition of mHtt may bring exciting new directions in HD research, a few key questions need to be answered to understand its pathological relevance.

First, it is crucial to know whether phase transition is a universal mechanism underlying mHtt aggregation in all cells, especially in neurons. Studies so far were carried out *in vitro*, in yeast cells and in HEK cells. It is critical to confirm the current findings in more relevant HD cell models such as neuronal cell lines or iPSC-derived neurons (Geater et al., 2018). Cell-type-specific difference of mHtt aggregation has been reported in mice HD models (Jansen et al., 2017). Microglia from frontal cortex and striatum have much lower frequency of nuclear mHtt aggregation than neurons and other glia cells from the same brain areas, despite of similar mHtt expression levels. The still unclear mechanisms underlying the cell-type-specific mHtt aggregation might also affect mHtt phase transition.

Second, mHtt is known to form aggregates in both the cytoplasm and nucleus, which are of very different biomolecule environment. Moreover, it is proposed that cytosolic and nuclear mHtt aggregates contribute differently to toxicity (Jimenez-Sanchez et al., 2017). It is thus necessary to compare cytosolic and nuclear mHtt phase transition, if applicable, to understand their physiological consequences. For example, HD is known to manifest nucleolar dysfunction and impaired rDNA transcription (Lee et al., 2014). Very recently, Frottin et al. found that the nucleolar granular component (GC) also undergoes phase separation and can serve as a membrane-less protein quality control compartment to temporarily store denatured proteins and prevent them from irreversible aggregation (Frottin et al., 2019). But the capacity of the nucleolus to buffer proteinaceous folding stress is limited. When overloaded, the GC liquid phase will go through a transition to solid phase and subsequently lead to nucleolar dysfunction. Whether intranuclear mHtt affect GC phase separation, for instance, by disrupting the process directly or by overwhelming its buffering capacity, will be a very interesting question worth testing.

Third, according to the reports so far, the expanded polyQ is required for mHtt to form solid-like assemblies but not for some liquid like assemblies. In other words, wild-type Htt (wtHtt) can also form liquid-like assemblies. Considering most HD patients are heterozygous that expresses a mixture of wtHtt and mHtt, it is closely relevant to study possible interactions between wtHtt and mHtt in their phase transition. The interaction between wtHtt and mHtt has been reported to affect disease progression and mHtt toxicity (Ho et al., 2001; Leavitt et al., 2001; Aziz et al., 2009; Saleh et al., 2014). Htt with wide-type polyQ length (25Q) was also found in mHtt (103Q) aggregates (Duennwald et al., 2006). Whether these reported interactions involve phase transition will be another interesting question to explore.

In summary, the discovery of phase transition of mHtt revealed a new mechanism involved in mHtt aggregation. Understanding the pathological relevance of different mHtt phases (e.g., the cytotoxicity of liquid phase and solid phase) and the factors that affect their transition, such as profilin that stabilize soluble mHtt phase and destabilize mHtt aggregates, will be crucial in identifying potential targets to interfere mHtt phase transition. Antagonizing the pathological mHtt phase transitions and correcting the normal phase transition could be a new strategy in treating the disease.

## Future Perspectives

It’s been almost three decades since the discovery of the causing gene of HD and the identity of aggregates found in patients. However, the mechanisms of mHtt aggregation and toxicity are still not fully understood. The discovery of the phase transition of mHtt added a new mechanism of mHtt aggregation and will help us to have a better understanding of the underlying molecular events. Considering that Htt with regular polyQ length can also go through LLPS, it is possible that de-regulated phase transition of mHtt also contribute to the pathology of the disease.

Moreover, accumulating evidence indicate that phase transition is used by the cell to regulate diverse intracellular biological processes such as autophagy (Sun et al., 2018; Fujioka et al., 2020) and protein quality control (Frottin et al., 2019; Yasuda et al., 2020), both are compromised in HD. Can phase transition be a new mechanism that mHtt affect normal cellular “daily life”? In other words, is it possible that mHtt undermine essential cellular functions via disrupting their phase transition-mediated events? Furthermore, can the phase transition of mHtt interfere with phase transition of other biological macromolecules? Some hundreds of proteins were found in mHtt aggregates (Hosp et al., 2017), could some of them be a result of mHtt-disrupted phase transition? Given the case that profilin affects the phase boundaries of mHtt, this possibility looks conceivable and is certainly worth testing, at least for mHtt-interacting proteins.

Although intracellular phase transition was confirmed not for long, it has already been reported to be involved in a number of diseases features protein aggregation, including amyotrophic lateral sclerosis (ALS), frontotemporal dementia (FTD) (Murakami et al., 2015; Patel et al., 2015; Monahan et al., 2017), AD (Ambadipudi et al., 2017; Wegmann et al., 2018). and HD (this review). Phase transition thus may serve as a new targetable process for treating these diseases (Verdile et al., 2019). However, like the case of HD discussed in this review, this can only base on a thorough knowledge of phase separation in general and phase transition of the specific target protein.

## Author Contributions

JY and XY wrote, reviewed, and edited the article. All authors contributed to the article and approved the submitted version.

## Conflict of Interest

The authors declare that the research was conducted in the absence of any commercial or financial relationships that could be construed as a potential conflict of interest.

## References

[B1] Aktar F. Burudpakdee C. Polanco M. Pei S. Swayne T. C. Lipke P. N. (2019). The huntingtin inclusion is a dynamic phase-separated compartment. *Life Sci. Alliance* 2:e201900489. 10.26508/lsa.201900489 31527136 PMC6749095

[B2] Alberti S. (2017). Phase separation in biology. *Curr. Biol.* 27 R1097–R1102. 10.1016/j.cub.2017.08.069 29065286

[B3] Ambadipudi S. Biernat J. Riedel D. Mandelkow E. Zweckstetter M. (2017). Liquid-liquid phase separation of the microtubule-binding repeats of the Alzheimer-related protein Tau. *Nat. Commun.* 8:275. 10.1038/s41467-017-00480-0 28819146 PMC5561136

[B4] Andre W. Sandt C. Dumas P. Djian P. Hoffner G. (2013). Structure of inclusions of Huntington’s disease brain revealed by synchrotron infrared microspectroscopy: polymorphism and relevance to cytotoxicity. *Anal. Chem.* 85 3765–3773. 10.1021/ac400038b 23458159

[B5] Arrasate M. Finkbeiner S. (2012). Protein aggregates in Huntington’s disease. *Exp. Neurol.* 238 1–11. 10.1016/j.expneurol.2011.12.013 22200539 PMC3909772

[B6] Arrasate M. Mitra S. Schweitzer E. S. Segal M. R. Finkbeiner S. (2004). Inclusion body formation reduces levels of mutant huntingtin and the risk of neuronal death. *Nature* 431 805–810. 10.1038/nature02998 15483602

[B7] Ast A. Buntru A. Schindler F. Hasenkopf R. Schulz A. Brusendorf L. (2018). mHTT seeding activity: a marker of disease progression and neurotoxicity in models of Huntington’s Disease. *Mol. Cell* 71 675–688e6. 10.1016/j.molcel.2018.07.032 30193095

[B8] Aziz N. A. Jurgens C. K. Landwehrmeyer G. B. Group E. R. S. van Roon-Mom W. M. C. van Ommen G. J. B. (2009). Normal and mutant HTT interact to affect clinical severity and progression in Huntington disease. *Neurology* 73 1280–1285. 10.1212/WNL.0b013e3181bd1121 19776381

[B9] Becher M. W. Kotzuk J. A. Sharp A. H. Davies S. W. Bates G. P. Price D. L. (1998). Intranuclear neuronal inclusions in Huntington’s disease and dentatorubral and pallidoluysian atrophy: correlation between the density of inclusions and IT15 CAG triplet repeat length. *Neurobiol. Dis.* 4 387–397. 10.1006/nbdi.1998.0168 9666478

[B10] Boeynaems S. Alberti S. Fawzi N. L. Mittag T. Polymenidou M. Rousseau F. (2018). Protein phase separation: a new phase in cell biology. *Trends Cell Biol.* 28 420–435. 10.1016/j.tcb.2018.02.004 29602697 PMC6034118

[B11] Brangwynne C. P. Eckmann C. R. Courson D. S. Rybarska A. Hoege C. Gharakhani J. (2009). Germline P granules are liquid droplets that localize by controlled dissolution/condensation. *Science* 324 1729–1732. 10.1126/science.1172046 19460965

[B12] Brangwynne C. P. Mitchison T. J. Hyman A. A. (2011). Active liquid-like behavior of nucleoli determines their size and shape in Xenopus laevis oocytes. *Proc. Natl. Acad. Sci. U.S.A.* 108 4334–4339. 10.1073/pnas.1017150108 21368180 PMC3060270

[B13] Cable J. Brangwynne C. Seydoux G. Cowburn D. Pappu R. V. Castaneda C. A. (2019). Phase separation in biology and disease-a symposium report. *Ann. N. Y. Acad. Sci.* 1452 3–11. 10.1111/nyas.14126 31199001 PMC6751006

[B14] Caron N. S. Hung C. L. Atwal R. S. Truant R. (2014). Live cell imaging and biophotonic methods reveal two types of mutant huntingtin inclusions. *Hum. Mol. Genet.* 23 2324–2338. 10.1093/hmg/ddt625 24334607

[B15] Davies S. W. Turmaine M. Cozens B. A. DiFiglia M. Sharp A. H. Ross C. A. (1997). Formation of neuronal intranuclear inclusions underlies the neurological dysfunction in mice transgenic for the HD mutation. *Cell* 90 537–548. 10.1016/s0092-8674(00)80513-9 9267033

[B16] DiFiglia M. Sapp E. Chase K. O. Davies S. W. Bates G. P. Vonsattel J. P. (1997). Aggregation of huntingtin in neuronal intranuclear inclusions and dystrophic neurites in brain. *Science* 277 1990–1993. 10.1126/science.277.5334.1990 9302293

[B17] Duennwald M. L. Jagadish S. Giorgini F. Muchowski P. J. Lindquist S. (2006). A network of protein interactions determines polyglutamine toxicity. *Proc. Natl. Acad. Sci. U.S.A.* 103 11051–11056. 10.1073/pnas.0604548103 16832049 PMC1544172

[B18] Frottin F. Schueder F. Tiwary S. Gupta R. Korner R. Schlichthaerle T. (2019). The nucleolus functions as a phase-separated protein quality control compartment. *Science* 365 342–347. 10.1126/science.aaw9157 31296649

[B19] Fu Y. Wu P. Pan Y. Sun X. Yang H. Difiglia M. (2017). A toxic mutant huntingtin species is resistant to selective autophagy. *Nat. Chem. Biol.* 13 1152–1154. 10.1038/nchembio.2461 28869595

[B20] Fujioka Y. Alam J. M. Noshiro D. Mouri K. Ando T. Okada Y. (2020). Phase separation organizes the site of autophagosome formation. *Nature* 578 301–305. 10.1038/s41586-020-1977-6 32025038

[B21] Geater C. Hernandez S. Thompson L. Mattis V. B. (2018). Cellular models: HD patient-derived pluripotent stem cells. *Methods Mol. Biol.* 1780 41–73. 10.1007/978-1-4939-7825-0_4 29856014

[B22] Gerlich D. W. (2017). Cell organization by liquid phase separation. *Nat. Rev. Mol. Cell Biol.* 18:593. 10.1038/nrm.2017.93 28875993

[B23] Gutekunst C. A. Li S. H. Yi H. Mulroy J. S. Kuemmerle S. Jones R. (1999). Nuclear and neuropil aggregates in Huntington’s disease: relationship to neuropathology. *J. Neurosci.* 19 2522–2534. 10.1523/jneurosci.19-07-02522.1999 10087066 PMC6786077

[B24] Ho L. W. Brown R. Maxwell M. Wyttenbach A. Rubinsztein D. C. (2001). Wild type Huntingtin reduces the cellular toxicity of mutant Huntingtin in mammalian cell models of Huntington’s disease. *J. Med. Genet.* 38 450–452. 10.1136/jmg.38.7.450 11432963 PMC1757193

[B25] Hosp F. Gutierrez-Angel S. Schaefer M. H. Cox J. Meissner F. Hipp M. S. (2017). spatiotemporal proteomic profiling of Huntington’s Disease inclusions reveals widespread loss of protein function. *Cell Rep.* 21 2291–2303. 10.1016/j.celrep.2017.10.097 29166617 PMC5714591

[B26] Hyman A. A. Weber C. A. Julicher F. (2014). Liquid-liquid phase separation in biology. *Annu. Rev. Cell Dev. Biol.* 30 39–58. 10.1146/annurev-cellbio-100913-013325 25288112

[B27] Iuchi S. Hoffner G. Verbeke P. Djian P. Green H. (2003). Oligomeric and polymeric aggregates formed by proteins containing expanded polyglutamine. *Proc. Natl. Acad. Sci. U.S.A.* 100 2409–2414. 10.1073/pnas.0437660100 12591956 PMC151354

[B28] Jansen A. H. van Hal M. Op den Kelder I. C. Meier R. T. de Ruiter A. A. Schut M. H. (2017). Frequency of nuclear mutant huntingtin inclusion formation in neurons and glia is cell-type-specific. *Glia* 65 50–61. 10.1002/glia.23050 27615381 PMC5129569

[B29] Jeon I. Cicchetti F. Cisbani G. Lee S. Li E. Bae J. (2016). Human-to-mouse prion-like propagation of mutant huntingtin protein. *Acta Neuropathol.* 132 577–592. 10.1007/s00401-016-1582-9 27221146 PMC5023734

[B30] Jimenez-Sanchez M. Licitra F. Underwood B. R. Rubinsztein D. C. (2017). Huntington’s disease: mechanisms of pathogenesis and therapeutic strategies. *Cold Spring Harb. Perspect. Med.* 7:a024240. 10.1101/cshperspect.a024240 27940602 PMC5495055

[B31] Kim M. Lee H. S. LaForet G. McIntyre C. Martin E. J. Chang P. (1999). Mutant huntingtin expression in clonal striatal cells: dissociation of inclusion formation and neuronal survival by caspase inhibition. *J. Neurosci.*. 19 964–973. 10.1523/jneurosci.19-03-00964.1999 9920660 PMC6782141

[B32] Kim Y. E. Hosp F. Frottin F. Ge H. Mann M. Hayer-Hartl M. (2016). Soluble oligomers of PolyQ-expanded huntingtin target a multiplicity of key cellular factors. *Mol. Cell* 63 951–964. 10.1016/j.molcel.2016.07.022 27570076

[B33] Krobitsch S. Lindquist S. (2000). Aggregation of huntingtin in yeast varies with the length of the polyglutamine expansion and the expression of chaperone proteins. *Proc. Natl. Acad. Sci. U.S.A.* 97 1589–1594. 10.1073/pnas.97.4.1589 10677504 PMC26479

[B34] Lajoie P. Snapp E. L. (2010). Formation and toxicity of soluble polyglutamine oligomers in living cells. *PLoS One* 5:e15245. 10.1371/journal.pone.0015245 21209946 PMC3011017

[B35] Leavitt B. R. Guttman J. A. Hodgson J. G. Kimel G. H. Singaraja R. Vogl A. W. (2001). Wild-type huntingtin reduces the cellular toxicity of mutant huntingtin in vivo. *Am. J. Hum. Genet.* 68 313–324. 10.1086/318207 11133364 PMC1235265

[B36] Lee J. Hwang Y. J. Ryu H. Kowall N. W. Ryu H. (2014). Nucleolar dysfunction in Huntington’s disease. *Biochim. Biophys. Acta* 1842 785–790. 10.1016/j.bbadis.2013.09.017 24184605 PMC3972370

[B37] Legleiter J. Mitchell E. Lotz G. P. Sapp E. Ng C. DiFiglia M. (2010). Mutant huntingtin fragments form oligomers in a polyglutamine length-dependent manner in vitro and in vivo. *J. Biol. Chem.* 285 14777–14790. 10.1074/jbc.M109.093708 20220138 PMC2863238

[B38] MacDonald M. E. Ambrose C. M. Duyao M. P. Myers R. H. Lin C. Srinidhi L. (1993). A novel gene containing a trinucleotide repeat that is expanded and unstable on Huntington’s disease chromosomes. *Cell* 72 971–983. 10.1016/0092-8674(93)90585 8458085

[B39] Marcellin D. Abramowski D. Young D. Richter J. Weiss A. Marcel A. (2012). Fragments of HdhQ150 mutant huntingtin form a soluble oligomer pool that declines with aggregate deposition upon aging. *PLoS One* 7:e44457. 10.1371/journal.pone.0044457 22984513 PMC3440421

[B40] Meriin A. B. Zhang X. He X. Newnam G. P. Chernoff Y. O. Sherman M. Y. (2002). Huntington toxicity in yeast model depends on polyglutamine aggregation mediated by a prion-like protein Rnq1. *J. Cell Biol.* 157 997–1004. 10.1083/jcb.200112104 12058016 PMC2174031

[B41] Miller J. Arrasate M. Brooks E. Libeu C. P. Legleiter J. Hatters D. (2011). Identifying polyglutamine protein species in situ that best predict neurodegeneration. *Nat. Chem. Biol.* 7 925–934. 10.1038/nchembio.694 22037470 PMC3271120

[B42] Monahan Z. Ryan V. H. Janke A. M. Burke K. A. Rhoads S. N. Zerze G. H. (2017). Phosphorylation of the FUS low-complexity domain disrupts phase separation, aggregation, and toxicity. *EMBO J.* 36 2951–2967. 10.15252/embj.201696394 28790177 PMC5641905

[B43] Murakami T. Qamar S. Lin J. Q. Schierle G. S. Rees E. Miyashita A. (2015). ALS/FTD mutation-induced Phase transition of FUS liquid droplets and reversible hydrogels into irreversible hydrogels impairs RNP granule function. *Neuron* 88 678–690. 10.1016/j.neuron.2015.10.030 26526393 PMC4660210

[B44] Olshina M. A. Angley L. M. Ramdzan Y. M. Tang J. Bailey M. F. Hill A. F. (2010). Tracking mutant huntingtin aggregation kinetics in cells reveals three major populations that include an invariant oligomer pool. *J. Biol. Chem.* 285 21807–21816. 10.1074/jbc.M109.084434 20444706 PMC2898425

[B45] Ordway J. M. Tallaksen-Greene S. Gutekunst C. A. Bernstein E. M. Cearley J. A. Wiener H. W. (1997). Ectopically expressed CAG repeats cause intranuclear inclusions and a progressive late onset neurological phenotype in the mouse. *Cell* 91 753–763. 10.1016/s0092-8674(00)80464-x 9413985

[B46] Patel A. Lee H. O. Jawerth L. Maharana S. Jahnel M. Hein M. Y. (2015). A liquid-to-solid phase transition of the ALS protein FUS accelerated by disease mutation. *Cell* 162 1066–1077. 10.1016/j.cell.2015.07.047 26317470

[B47] Pearce M. P. M. Kopito R. R. (2018). Prion-like characteristics of polyglutamine-containing proteins. *Cold Spring Harb. Perspect. Med.* 8:a024257. 10.1101/cshperspect.a024257 28096245 PMC5793740

[B48] Pecho-Vrieseling E. Rieker C. Fuchs S. Bleckmann D. Esposito M. S. Botta P. (2014). Transneuronal propagation of mutant huntingtin contributes to non-cell autonomous pathology in neurons. *Nat. Neurosci.* 17 1064–1072. 10.1038/nn.3761 25017010

[B49] Perutz M. F. Windle A. H. (2001). Cause of neural death in neurodegenerative diseases attributable to expansion of glutamine repeats. *Nature* 412 143–144. 10.1038/35084141 11449262

[B50] Peskett T. R. Rau F. O’Driscoll J. Patani R. Lowe A. R. Saibil H. R. (2018). A liquid to solid phase transition underlying pathological huntingtin Exon1 aggregation. *Mol. Cell* 70 588–601e6. 10.1016/j.molcel.2018.04.007 29754822 PMC5971205

[B51] Peters-Libeu C. Miller J. Rutenber E. Newhouse Y. Krishnan P. Cheung K. (2012). Disease-associated polyglutamine stretches in monomeric huntingtin adopt a compact structure. *J. Mol. Biol.* 421 587–600. 10.1016/j.jmb.2012.01.034 22306738 PMC3358578

[B52] Poirier M. A. Li H. Macosko J. Cai S. Amzel M. Ross C. A. (2002). Huntingtin spheroids and protofibrils as precursors in polyglutamine fibrilization. *J. Biol. Chem.* 277 41032–41037. 10.1074/jbc.M205809200 12171927

[B53] Posey A. E. Ruff K. M. Harmon T. S. Crick S. L. Li A. Diamond M. I. (2018). Profilin reduces aggregation and phase separation of huntingtin N-terminal fragments by preferentially binding to soluble monomers and oligomers. *J. Biol. Chem.* 293 3734–3746. 10.1074/jbc.RA117.000357 29358329 PMC5846159

[B54] Ramdzan Y. M. Trubetskov M. M. Ormsby A. R. Newcombe E. A. Sui X. Tobin M. J. (2017). Huntingtin inclusions trigger cellular quiescence, deactivate apoptosis, and lead to delayed necrosis. *Cell Rep.* 19 919–927. 10.1016/j.celrep.2017.04.029 28467905

[B55] Saleh A. A. Bhadra A. K. Roy I. (2014). Cytotoxicity of mutant huntingtin fragment in yeast can be modulated by the expression level of wild type huntingtin fragment. *ACS Chem. Neurosci.* 5 205–215. 10.1021/cn400171d 24377263 PMC3963126

[B56] Saudou F. Finkbeiner S. Devys D. Greenberg M. E. (1998). Huntingtin acts in the nucleus to induce apoptosis but death does not correlate with the formation of intranuclear inclusions. *Cell* 95 55–66. 10.1016/s0092-8674(00)81782-1 9778247

[B57] Scherzinger E. Lurz R. Turmaine M. Mangiarini L. Hollenbach B. Hasenbank R. (1997). Huntingtin-encoded polyglutamine expansions form amyloid-like protein aggregates in vitro and in vivo. *Cell* 90 549–558. 10.1016/s0092-8674(00)80514-0 9267034

[B58] Scherzinger E. Sittler A. Schweiger K. Heiser V. Lurz R. Hasenbank R. (1999). Self-assembly of polyglutamine-containing huntingtin fragments into amyloid-like fibrils: implications for Huntington’s disease pathology. *Proc. Natl. Acad. Sci. U.S.A.* 96 4604–4609. 10.1073/pnas.96.8.4604 10200309 PMC16379

[B59] Shin Y. Brangwynne C. P. (2017). Liquid phase condensation in cell physiology and disease. *Science* 357:eaaf4382. 10.1126/science.aaf4382 28935776

[B60] Spanic E. Langer Horvat L. Hof P. R. Simic G. (2019). Role of microglial cells in alzheimer’s disease tau propagation. *Front. Aging Neurosci.* 11:271. 10.3389/fnagi.2019.00271 31636558 PMC6787141

[B61] Sun D. Wu R. Zheng J. Li P. Yu L. (2018). Polyubiquitin chain-induced p62 phase separation drives autophagic cargo segregation. *Cell Res.* 28 405–415. 10.1038/s41422-018-0017-7 29507397 PMC5939046

[B62] Takahashi T. Kikuchi S. Katada S. Nagai Y. Nishizawa M. Onodera O. (2008). Soluble polyglutamine oligomers formed prior to inclusion body formation are cytotoxic. *Hum. Mol. Genet.* 17 345–356. 10.1093/hmg/ddm311 17947294

[B63] Verdile V. De Paola E. Paronetto M. P. (2019). Aberrant phase transitions: side effects and novel therapeutic strategies in human Disease. *Front. Genet.* 10:173. 10.3389/fgene.2019.00173 30967892 PMC6440380

[B64] Wegmann S. Eftekharzadeh B. Tepper K. Zoltowska K. M. Bennett R. E. Dujardin S. (2018). Tau protein liquid-liquid phase separation can initiate tau aggregation. *EMBO J* 37:e98049. 10.15252/embj.201798049 29472250 PMC5881631

[B65] Wilson E. B. (1899). The structure of protoplasm. *Science* 10 33–45. 10.1126/science.10.237.33 17829686

[B66] Yasuda S. Tsuchiya H. Kaiho A. Guo Q. Ikeuchi K. Endo A. (2020). Stress- and ubiquitylation-dependent phase separation of the proteasome. *Nature* 578 296–300. 10.1038/s41586-020-1982-9 32025036

